# Preparation and recyclable catalysis performance of functional macroporous polyHIPE immobilized with gold nanoparticles on its surface†

**DOI:** 10.1039/c8ra00089a

**Published:** 2018-02-06

**Authors:** Weizhong Yuan, Xiangnan Chen, Yifan Xu, Chuan Yan, Yang Liu, Weishuai Lian, Yun Zhou, Zhihong Li

**Affiliations:** Tongji Hospital, School of Medicine, Department of Interventional and Vascular Surgeery of Shanghai Tenth People's Hospital, School of Materials Science and Engineering, Tongji University Shanghai 201804 P. R. China yuanwz@tongji.edu.cn 25936615@qq.com lianweishuai@126.com Zhouyunsy@126.com; Division of General Surgery, Shanghai Pudong New District Zhoupu Hospital Shanghai 201200 P. R. China lance007@126.com

## Abstract

High internal phase emulsion polymerization was adopted for preparing macroporous polymeric monoliths, polyHIPE–Br (PHIPE–Br). Macroporous PHIPE–Br was used as the initiator to initiate the atom transfer radical polymerization (ATRP) of glycidyl methacrylate (GMA), resulting in a dense coating of polymers on the PHIPE surface and PHIPE–PGMA was obtained. Through a ring-opening addition reaction with TETA, a surface amino-polymer modified functional macroporous PHIPE–PGMA–TETA, was prepared conveniently. Gold nanoparticles could be easily *in situ* prepared and immobilized on the surface of PHIPE–PGMA–TETA. Characterization by scanning electron microscopy (SEM), EDX-mapping and TGA showed that PHIPE–PGMA–TETA was immobilized by the gold nanoparticles, and presented good catalytic properties. Moreover, the macroporous catalytic material, PHIPE–PGMA–TETA/Au NPs, presented recyclable catalytic performance without any decrease in activity. The materials and methods to form the monoliths and immobilize metal nanoparticles were simple and efficient, thus, opening new possibilities for highly porous PHIPE in catalysis applications.

## Abstract 2

The macroporous material PHIPE–PGMA–TETA/Au NPs has an open-cell and porous structure, and can effectively catalyze the reduction of 4-nitrophenol.

## Graphical abstract

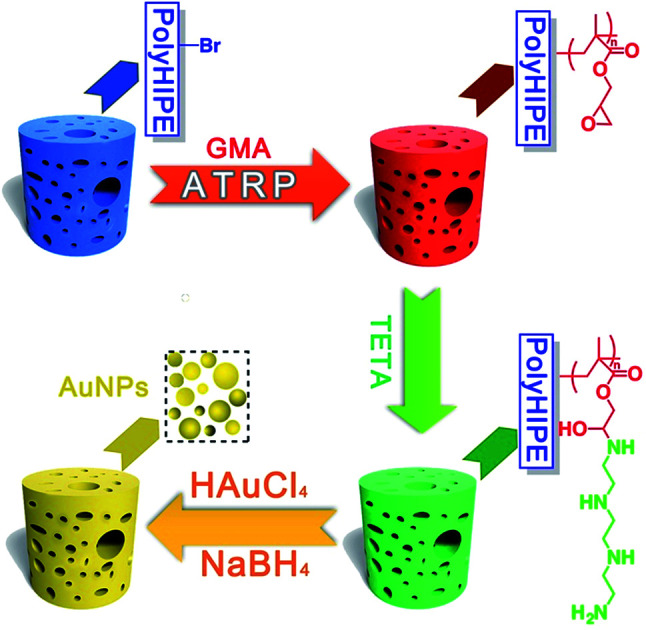

## Introduction

With the rapid development of nanotechnology, gold nanoparticles (Au NPs) have been widely used in fields ranging from optoelectronics, electrochemistry, biosensors, antibacterial applications to catalysis.^1–8^ The catalytic activity of gold nanoparticles is related to their size, distribution and morphology.^9–12^ How to obtain highly dispersed, size controllable, stable, easy to separate, long-term and recyclable gold nanoparticles catalysts is an important research goal, and also a bottleneck for its practical application. Ying *et al.* claimed in a review that the challenge in the future of nanocatalyst research lies in the rational design and development of multifunctional, robust, and recyclable nanocomposite catalysts.^13^

For the high activity of Au NPs, the surface should be of large area and be active; however, for good stability and recyclable Au NPs, the surface should be sufficiently passivated. In order to improve the stability and catalytic activity of gold nanoparticles, support of metal catalysts on other materials is favourable. The structure and properties of the support, and the interaction of the support and gold nanoparticles may improve the performance.^14,15^ Recently, macroporous polymeric matrixes are becoming popular supports for metal nanoparticles.^16–32^ For example, Sui *et al.* successfully immobilized catalytic palladium nanoparticles onto a cellulose sponge, and the resulting catalyst appears recyclable.^25^ Wan *et al.* successfully prepared a porous monolith to support recyclable Au NPs using a one-pot strategy.^27^ Zhang *et al.* report on a simple, broadly applicable method for depositing metal-nanoparticle films on a wide variety of solid surfaces under all aqueous conditions.^28^ Yoon Sung Nam *et al.* synthesize and immobilize metal nanoparticles on electrospun nanofibers and hybrid microspheres for recyclable catalysis.^33–36^ However, most of these preparation methods were relatively complex. Therefore, it is necessary to develop a simple, efficient and reusable metal nanoparticles catalytic system for the catalytic decomposition of trace toxic and harmful organic compound in water, *e.g.* 4-nitrophenol *etc.*

Macroporous polymeric matrixes combine properties of mass transport, excellent flow and a high surface area, which is ideally suited for a variety of applications including adsorption, catalysts and so on.^37–49^ High internal phase emulsions (HIPE) is such a kind of macroporous polymeric matrixes with high surface area and open-cellular structure. PolyHIPE is created by the polymerization of the continuous monomer-containing phase of a high internal phase emulsion (HIPE) with an internal phase volume of greater than 74%.^37^ The continuous phase is organic, and the dispersion phase is aqueous which mainly consists of microscale water droplets. After the polymerization of the continuous phase and removing the dispersion phase, the porous material polyHIPE with a shape determined by the reacting vessel was gained.^50,51^ Wan *et al.* successfully prepared a dendritic amphiphile mediated preparation of functional polyHIPE which can simultaneously eliminate anionic dyes, anionic surfactants and hydrophobic polycyclic aromatic hydrocarbons (PAHs) from water.^40^ Jing *et al.* successfully prepared a porous heterogeneous photocatalyst by HIPE polymerization, which demonstrated a very high catalytic efficiency and can be reused for photocatalyzed oxidation of thioanisole under visible light.^38^ Therefore, it is convenient to achieve a highly efficient and reusable catalytic system by immobilization of metal nanoparticles on the surface of polymer porous materials.

**Scheme 1:**
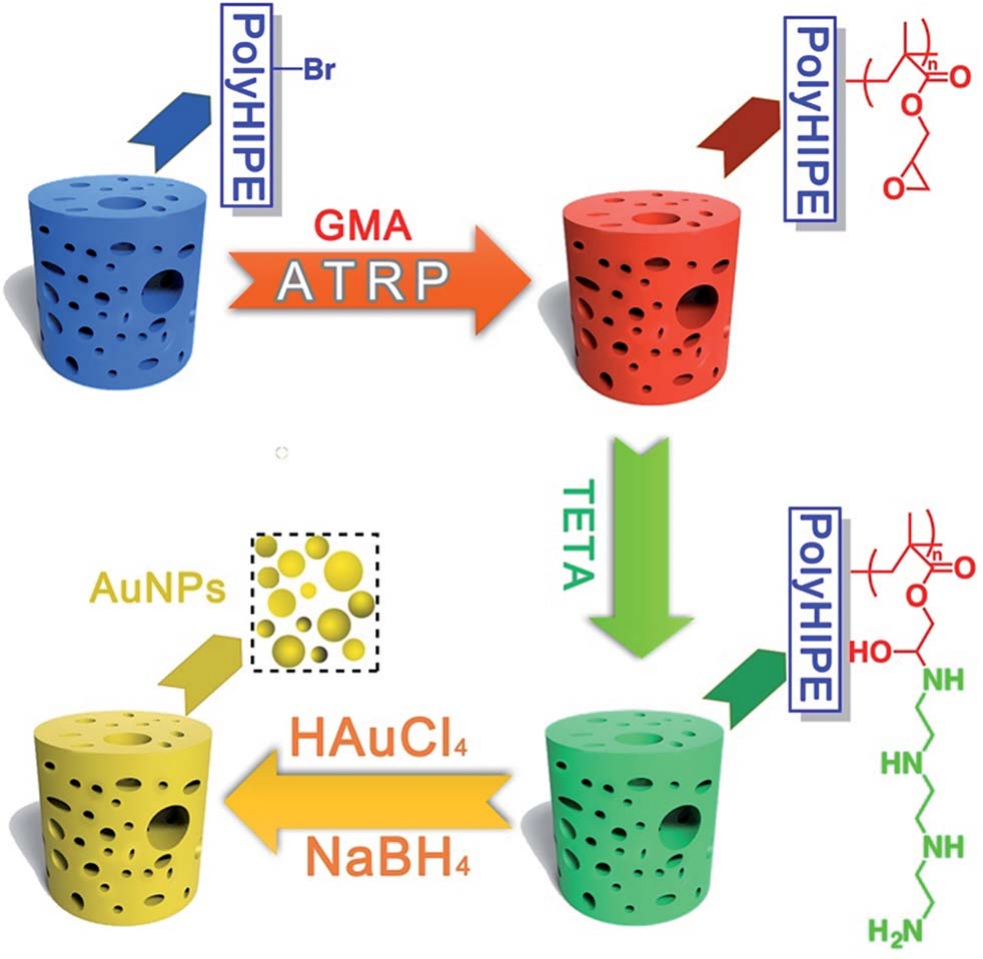
Synthetic pathway to PHIPE–PGMA, PHIPE–PGMA–TETA and PHIPE–PGMA–TETA/Au NPs.

Herein, in this work, high internal phase emulsion template method was adopted for preparing macroporous polymeric monoliths polyHIPE–Br. Through atom transfer radical polymerization (ATRP) of glycidyl methacrylate (GMA), and ring-opening addition reaction with TETA, a surface amino-polymers modified functional macroporous PHIPE–PGMA–TETA (PHIPE–PGMA–TETA), was prepared conveniently. Gold nanoparticles could be easily *in situ* prepared and immobilized on the surface of PHIPE–PGMA–TETA. The characterization of scanning electron microscopy (SEM), EDX-mapping and TGA shows that PHIPE–PGMA–TETA was immobilized with gold nanoparticles, and the catalytic properties were good. The catalytic materials, PHIPE–PGMA–TETA/Au NPs, are recyclable without any decrease in activity. As a result, PHIPE–PGMA–TETA/Au NPs is expected to be an efficient and reusable catalysis (Scheme 1).

## Experimental

### Materials

2-Hydroxyethyl acrylate, glycidyl methacrylate (GMA), 2-bromo-2-methylpropionyl bromide, triethylenetetramine (TETA) and divinylbenzene (DVB) were purchased from Aldrich and used as received. Styrene was passed through a column of basic alumina to remove inhibitor. Triethylamine, dichloromethane, tetrahydrofuran (THF) and dimethylfomamide (DMF) was dried by CaH_2_ and distilled under reduced pressure. Copper bromide (CuBr, Shanghai Chemical Reagent Co., 99%) was treated by stirring in glacial acetic acid and washed with ethanol several times. The other reagents and solvents were local commercial products and used without further purification. 2-Acryloxyethyl-2′-bromoisobutyrate was synthesized according to the literature with some modification.^52^

### Characterization

#### Attenuated total internal reflectance Fourier transform infrared (ATR FT-IR)

ATR FT-IR spectra of samples were recorded on an AVATAR 360 ESP FT-IR spectrometer (Thermo Nicolet, American).

#### Nuclear magnetic resonance (NMR)

^1^H NMR spectra of samples were obtained from a Bruker DMX-500 NMR spectrometer with CDCl_3_ as solvent. The chemical shifts were relative to tetramethylsilane.

#### Scanning electron microscopy (SEM)

The morphology of polyHIPE samples were observed with a FEI QUANTA250FEG microscope by placing a sample on an aluminum stub with an adhesive carbon pad. PolyHIPE samples without gold species were coated with gold. The samples were measured at an accelerating voltage of 10 kV and spot size of 10.0 nm. Energy dispersive X-ray spectroscopy (EDX) was measured along with the SEM. PolyHIPE samples were coated with platinum.

#### Mercury intrusion porosimetry (MIP)

The porosity and surface area of polyHIPE were measured with a mercury intrusion porosimetry (MIP) of Pore Master 60-GT (Quantachrome Corp).

#### Thermogravimetric analysis (TGA)

The TGA data were obtained with a heating rate of 10 K min^−1^ using a TA TGA-2950 apparatus.

#### Optical transmittances

UV-vis spectroscopy was performed on a UV-visible spectrophotometer (Lambda 35, PerkinElmer).

### Synthesis of 2-acryloxyethyl-2′-bromoisobutyrate

2-Hydroxyethyl acrylate (1.89 g, 16.3 mmol) and triethyl amine (1.65 g, 16.3 mmol) were added to a flask containing 100 mL of dry THF, stirred, and cooled to 0 °C. With use of an addition funnel, 2-bromo-2-methylpropionyl bromide (4.37 g, 19.0 mmol) was added dropwise. Upon completion of the addition, the reaction mixture was brought to room temperature and allowed to stand for 18 h. The product was extracted into ethyl acetate, dried over anhydrous MgSO_4_, filtered and reduced *in vacuo* to yield yellow oil.

^1^H NMR (300 MHz, CDCl_3_) *δ* (ppm) 6.40 (*CH*_2_ = CH–, 1H, dd), 6.09 (CH_2_ = *CH*–, 1H, dd), 5.85 (*CH*_2_ = CH–, 1H, dd), 4.40 (O–*CH*_2_–*CH*_2_–O–, 4H, s), 1.95 (–C–*CH*_3_–, 6H, s).

### Preparation of polyHIPE–Br (PHIPE–Br)

A total of styrene (0.85 g), divinylbenzene (0.35 g), 2-acryloxyethyl-2′-bromoisobutyrate (0.4 g) and Span 80 surfactant (0.4 g) were placed in a reactor. Then, the mixture was stirred using an overhead stirrer at 300 rpm. The aqueous solution was prepared by dissolving 0.6 g of potassium peroxodisulfate (K_2_S_2_O_8_) in 50 mL of deionized water. The aqueous solution (8 mL) was added dropwise with vigorous stirring. The resulting emulsion was transferred to a glass mold and heated at 70 °C for 24 h, followed by washing in sequence with ethanol, THF and acetone before drying in vacuum at 40 °C overnight.

ATR FT-IR (cm^−1^): 2826–3037 (*v*_C–H_), 1737 (*v*_C=O_).

### Preparation of PHIPE–PGMA

PHIPE–Br (100 mg), GMA (2.0 g, 14 mmol), THF (15 mL), CuBr (17.8 mg, 0.124 mmol) and PMDETA (21.5 mg, 0.124 mmol) were added to a Schlenk tube and subjected to three freeze–thaw cycles, then stirred at 65 °C under Ar atmosphere for 3.5 h. After being cooled to room temperature, the reaction tube was opened to air. The PHIPE–PGMA was then washed several times in THF for 12 h and acetone for 12 h using a Soxhlet extractor in order to remove the remaining monomers, the copper catalysts and PMDETA. After drying in vacuum, the resulting product was obtained (157.4 mg).

ATR FT-IR (cm^−1^): 2786–3041 (*v*_C–H_), 1737 (*v*_C=O_), 1263, 1157 (*v*_C–O–C_).

### Preparation of PHIPE–PGMA–TETA

The epoxy activated PHIPE–PGMA were immersed into a solution containing 250 g of 30% (w/w) triethylenetetramine (TETA) solution and 5 g of sodium carbonate. Then the reaction was carried out at 70 °C for 2 h under stirring for the amination of monoliths. Subsequently, the monoliths were separated from the solution and thoroughly rinsed with deionized water until neutral. After drying in vacuum, the resulting product was obtained (198.2 mg).

ATR FT-IR (cm^−1^): 3127–3712 (*v*_N–H_), 1737 (*v*_C=O_), 1650 (*v*_C–N_).

### *In situ* preparation and immobilization of gold nanoparticles on the surface of PHIPE–PGMA–TETA

100 mg of PHIPE–PGMA–TETA monolith was immersed in 20 mL of deionized water and 0.5 mL of 4 mM HAuCl_4_ aqueous solution was added; then, 2 mL of 0.05 M NaBH_4_ aqueous solution was added dropwise with stirring. The reaction lasted for 1 h. The final product was purified through washing with water three times and dried under vacuum oven until constant weight.

### Catalytic reduction of 4-nitrophenol to 4-aminophenol in an aqueous medium

A typical experiment for the catalytic reduction of 4-nitrophenol (4-NP) to 4-aminophenol (4-AnP) was carried out as follows: 4-NP aqueous solution (0.05 mmol), NaBH_4_ (0.01 mol) aqueous solution were mixed in a colorimetric tube. We introduced 80 mg of catalyst (containing 0.02 mmol of Au) into the mixture with vigorous stirring. The bright-yellow solution faded gradually as the catalytic reaction proceeded. The catalytic activity was determined by a UV-vis spectrophotometer with a decrease at 400 nm in UV-vis absorption and a simultaneous increase in the absorption at 300 nm, indicating the formation of 4-AnP.

## Results and discussion

### Preparation of PHIPE–PGMA–TETA immobilized with gold nanoparticles

**Fig. 1:**
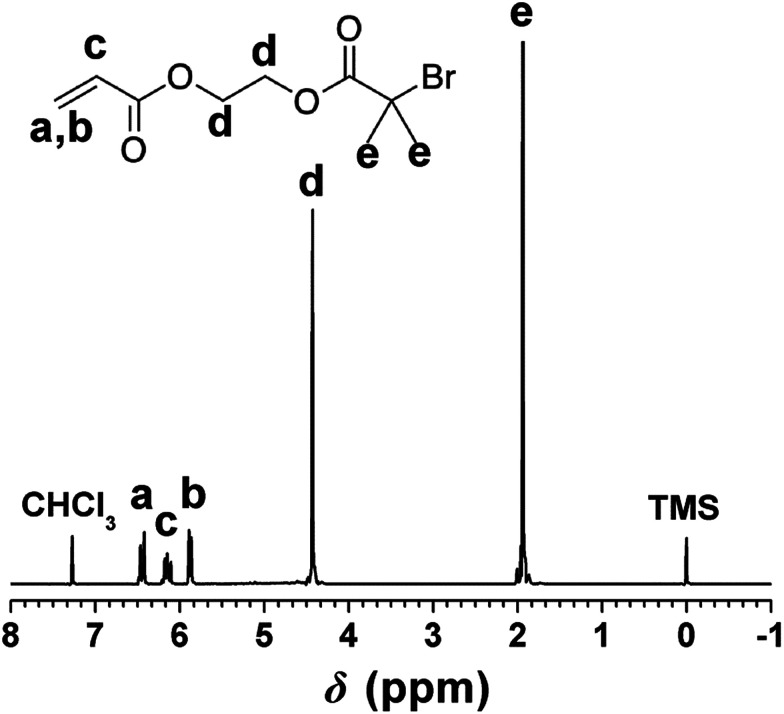
^1^H NMR spectrum of 2-acryloxyethyl-2′-bromoisobutyrate.

As the functional group of PHIPE–Br, 2-acryloxyethyl-2′-bromoisobutyrate was successfully synthesized by the reaction of 2-hydroxyethyl acrylate and 2-bromoisobutyryl bromide. The ^1^H NMR spectrum of 2-acryloxyethyl-2′-bromoisobutyrate was shown in Fig. 1. The absorption peaks at 6.40 ppm (a), 6.09 ppm (c), 5.85 ppm (b) were assigned to the protons on the vinyl group. The absorption peaks at 1.95 ppm (e) were the protons on the methyl group of 2-bromo-2-methylpropionyl bromide. The integral area ratio of peak a to peak e is 1 : 6. All these indicated that the 2-acryloxyethyl-2′-bromoisobutyrate was successfully synthesized.

GMA was frequently investigated for ATRP reaction and its ATRP method has been well developed. Macroporous PHIPE–Br were used as the initiators to initiate atom transfer radical polymerization (ATRP) of glycidyl methacrylate (GMA), resulting in a dense coating of epoxy groups on the polyHIPE surface. Epoxy groups could trigger ring-opening addition reaction with TETA. A surface amino-polymer modified functional macroporous PHIPE–PGMA–TETA could immobilize gold nanoparticles.

To investigate the graft ratio, the samples were weighed before and after the graft polymerization with GMA. The graft ratio *G* (wt%), was calculated according to
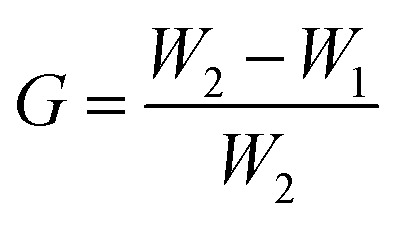
where *W*_1_ (mg) is the weight of PHIPE–Br macroinitiator and *W*_2_ (mg) is the weight of the PHIPE–PGMA sample.

From the difference of the PHIPE mass before and after GMA polymerization, around 36 wt% of grafted PGMA was estimated. The efficiency of click reaction of PHIPE–PGMA and TETA was about 69.2%, according to the PHIPE mass before and after the click reaction.

**Fig. 2:**
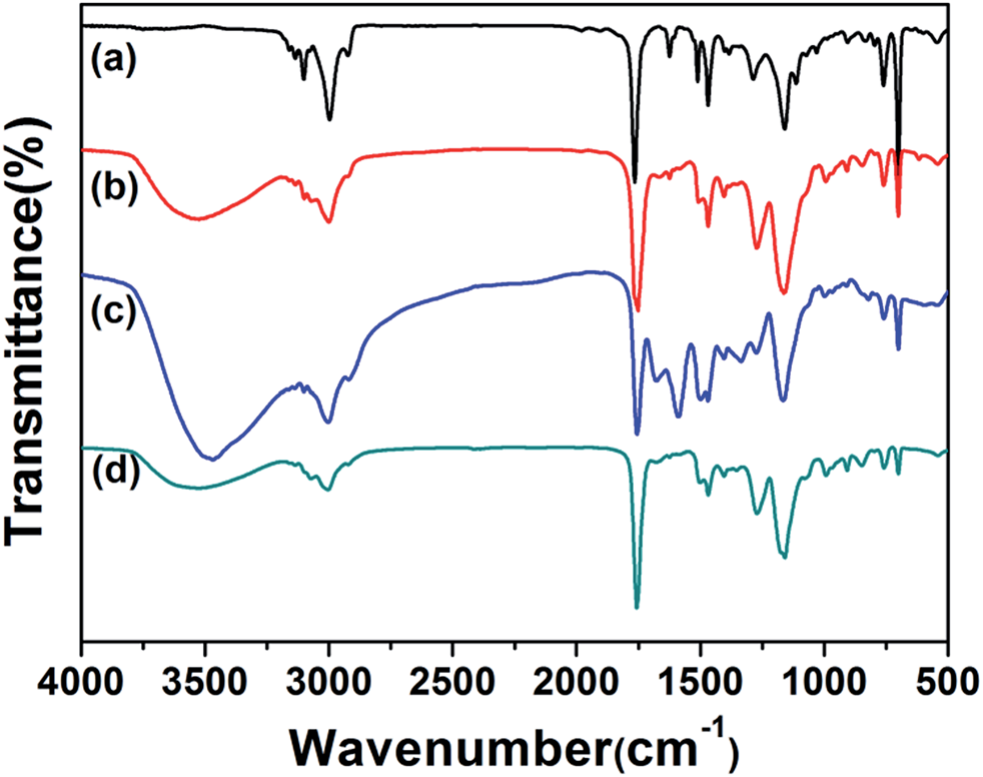
ATR FT-IR spectra of (a) PHIPE–Br, (b) PHIPE–PGMA, (c) PHIPE–PGMA–TETA and (d) PHIPE–PGMA–TETA/Au NPs.

The structures of PHIPE–Br, PHIPE–PGMA, PHIPE–PGMA–TETA and PHIPE–PGMA–TETA/Au NPs were also characterized by ATR FT-IR spectra, as shown in Fig. 2. For PHIPE–Br, the adsorptions at 1737 cm^−1^ should be assigned to the ester carbonyl group, as shown in Fig. 2(a). For PHIPE–PGMA, the adsorption at about 1263 cm^−1^ and 1157 cm^−1^ were significantly enhanced, which should be assigned to the epoxy group (Fig. 2(b)). These adsorptions indicated the successful grafting of the PGMA onto the surface of PHIPE. From ATR FT-IR spectrum of PHIPE–PGMA–TETA (Fig. 2(c)), the success of this functionalization was evident from the appearance of a new vibration band in the ATR FT-IR spectrum at 1650 cm^−1^, corresponding to the formation of the amide bond. The broad adsorption at about 3384 cm^−1^ belonged to the stretching vibration of the –NH groups. For PHIPE–PGMA–TETA/Au NPs, the adsorptions at 1650 cm^−1^ and 3384 cm^−1^ were significantly weakened (Fig. 2(d)). This was due to the presence of gold nanoparticles on the surface of the amino group.

**Fig. 3:**
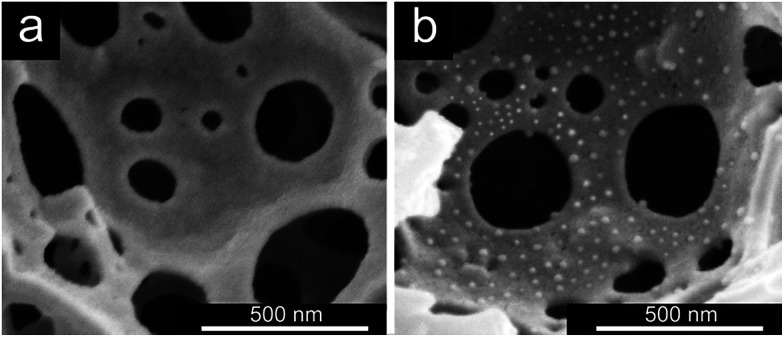
SEM images of (a) PHIPE–Br, (b) PHIPE–PGMA–TETA/Au NPs.

Further evidence for the successful grafting of the PGMA–TETA/Au NPs onto the surface of PHIPE was obtained from SEM images (Fig. 3). Fig. 3(a) was the SEM image of PHIPE–Br, and Fig. 3(b) was PHIPE–PGMA–TETA/Au NPs. It can be seen that the surface roughness could be observed and the divots presented in the blank sample were surrounded by smaller mounds. The change in surface morphology should be attributed to the presence of polymers and nanoparticles on the surface. Besides, in the process of *in situ* reduction, gold nanoparticles were easily aggregated. Therefore, some white particles on the surface of PHIPE–PGMA–TETA could be found in Fig. 3(b). The size of the gold nanoparticles is about 10.4 nm. The gold deposition has little effect on the pore properties except for the increase of density (Fig. S2, Table S1, ESI†).

**Fig. 4:**
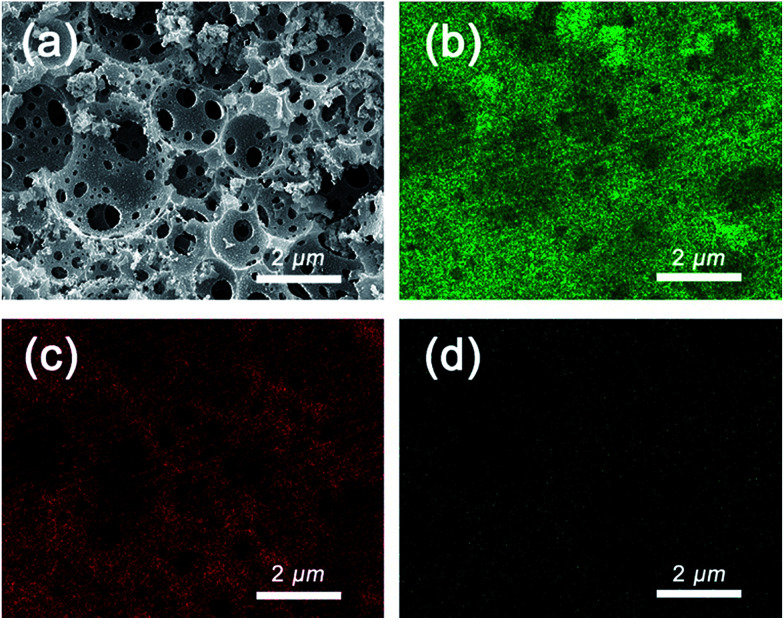
SEM of (a) PHIPE–GMA–TETA/Au NPs and the corresponding EDX micrographs of element (b) Au, (c) carbon and (d) oxygen on the surface layer. The detection depth of EDX is 2 μm and the scale bar is 2 μm.

To further explore the distribution of Au NPs, the sample was subjected to energy-dispersive X-ray spectroscopy (EDX). The sample PHIPE–PGMA–TETA/Au NPs were coated with platinum. Fig. 4(a) was the SEM of PHIPE–PGMA–TETA/Au NPs. Fig. 4(b) was the corresponding EDX micrographs of element Au on the surface layer, while Fig. 4(c) was of element carbon and Fig. 4(d) was oxygen. Elemental mapping images of Fig. 4(b and c) show no obvious difference between the distribution of Au and carbon, indicating that the Au NPs were homogeneously dispersed onto the surface of PHIPE–PGMA–TETA. The content of oxygen on the surface of PHIPE–PGMA–TETA/Au NPs was not as much as that of the Au and carbon elements, so the point of Fig. 4(d) was relatively sparse. These results proved the efficient immobilization of the PGMA–TETA brush for the resultant gold metallic nanoparticles with the aid of the coordination between the amino groups with gold atoms.

**Fig. 5:**
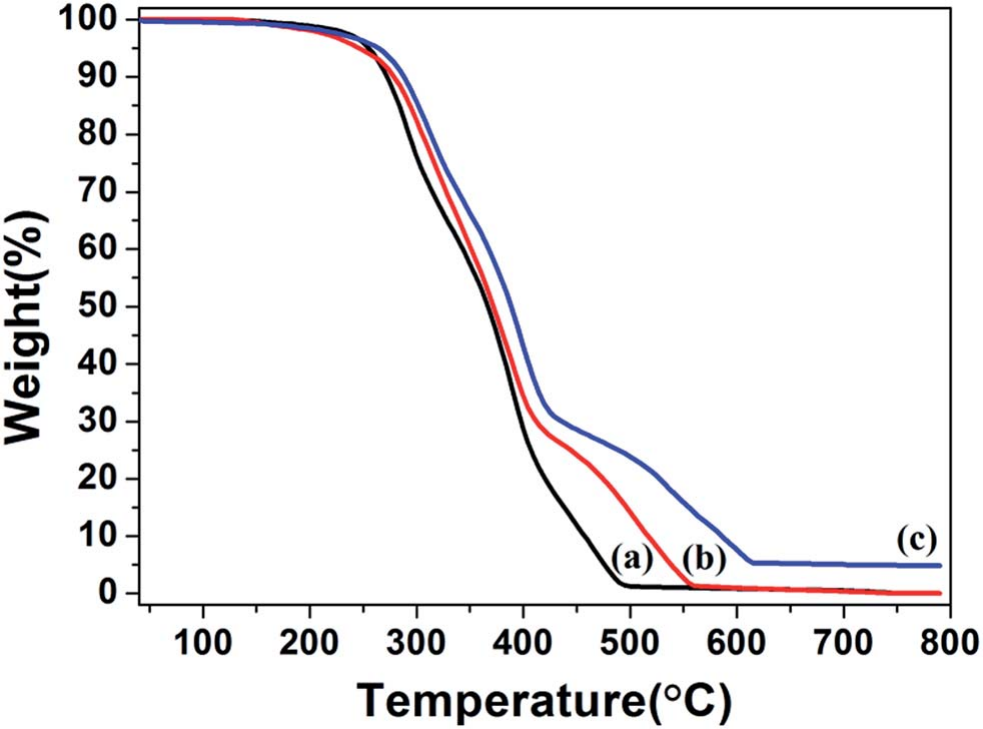
TGA curves of (a) PHIPE–PGMA, (b) PHIPE–PGMA–TETA and (c) PHIPE–PGMA–TETA/Au NPs (under air).

TGA measurements were determined for PHIPE–PGMA, PHIPE–PGMA–TETA and PHIPE–PGMA–TETA/Au NPs for further demonstration of the whole polymerization process as well as the surface modification. As shown in Fig. 5(a and b), the weight loss for the PHIPE–PGMA and PHIPE–PGMA–TETA were almost 100%. The major weight loss of these two PHIPEs from 250 to 500 °C was due to the decomposition of the polymer component. The TGA curve of PHIPE–PGMA–TETA/Au NPs was 95.2% with two distinct weight loss stages between 250 and 600 °C, which implied that ∼4.8% of Au NPs were efficiently coated onto the PHIPE polymeric matrixes. All of these results demonstrated that PHIPE–PGMA–TETA could immobilize the gold nanoparticles.

### Catalytic performance and recyclable property of PHIPE–PGMA–TETA/Au NPs

Because the outer PGMA–TETA brush in PHIPE–PGMA–TETA contained abundant amino groups as a good carrier for metallic nanoparticles, the gold nanoparticles were deposited onto the PHIPE–PGMA–TETA surface by the *in situ* reduction of HAuCl_4_ with the NaBH_4_ as reductant with the immobilization of Au nanoparticles from the efficient coordination effect.

The catalytic performance of PHIPE–PGMA–TETA/Au NPs is tested for the catalytic reduction of 4-NP to 4-AnP with NaBH_4_ as the reductant, which was widely used for testing the activation of the catalyst,^53^ and the catalytic mechanism in the supported gold nanoparticles was well studied in the literatures.^54–56^ Typically, when 4-NP (0.05 mmol) in water is mixed with NaBH_4_ (200 eq. of 4-NP) with stirring, a strong absorbance appears at 400 nm (due to the formation of anionic 4-NP) and the absorbance shows no decrease with time.

**Fig. 6:**
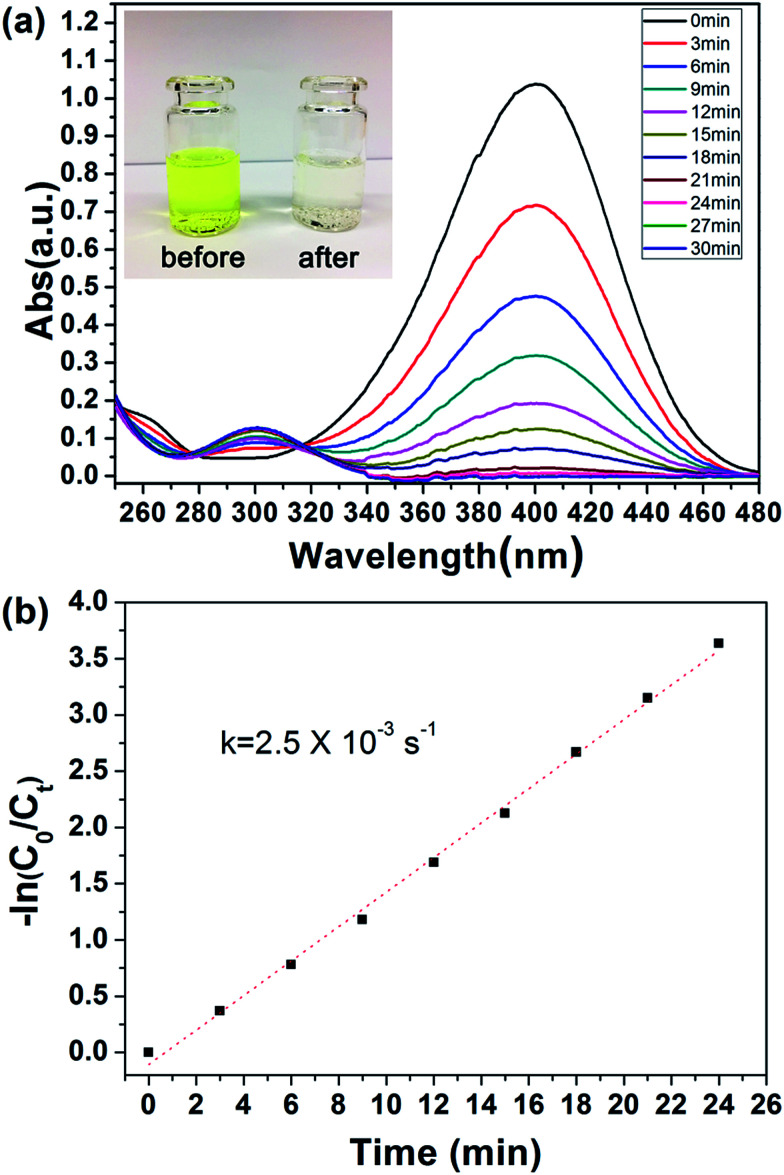
(a) UV/vis spectra during the reduction of 4-NP and (b) kinetic analysis of the reaction. Conditions: 4-NP (0.05 mmol), NaBH_4_ (200 eq.) and PHIPE–PGMA–TETA/Au NPs (0.4 eq. of Au atoms with respect to 4-NP).

After the introduction of PHIPE–PGMA–TETA/Au NPs (Au species is 0.4 eq. of 4-NP) into the reaction system, the reduction was performed quickly, as shown in Fig. 6(a). The absorption peak at 400 nm, decreased gradually and disappeared completely after 24 min. The new adsorption peak at 300 nm was simultaneously increased with proceeding of the reaction. It meant that the 4-NP was reduced to 4-AnP in the presence of PHIPE–PGMA–TETA/Au NPs catalyst.

Regarding the mechanism, it is generally believed that both reactants are adsorbed by the Au NPs (Langmuir–Hinshelwood model), followed by the reduction reaction proceeds and is at last the desorption of the reduced product.^56,57^ In case that the NaBH_4_ (200 eq. of 4-NP) is of much higher concentration, the kinetics can be considered as a pseudo-first-order reaction:ln(*C*_*t*_/*C*_0_) = ln(*A*_*t*_/*A*_0_) = −*k*_app_*t*where *C*_*t*_ and *C*_0_ represent the conversion at *t* moment and initial moment, *A*_*t*_ and *A*_0_ represent the absorbance at *t* moment and initial moment, and *k*_app_ represents the apparent rate constant. As shown in Fig. 6(b), the reduction kinetics complies with this model and the rate constant is 2.5 × 10^−3^ s^−1^ for the first cycle. Turnover frequency (TOF), which is defined as the total number of 4-NP molecules that the catalyst with a unit mole reduces per unit time.
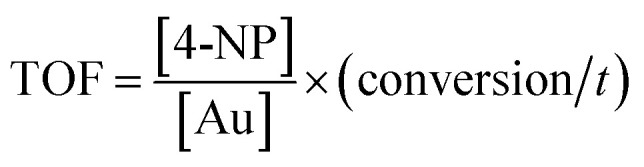


The TOF of PHIPE–PGMA–TETA/Au NPs is derived to be 6.3 h^−1^, which is equivalent to other supported catalysts.

**Fig. 7:**
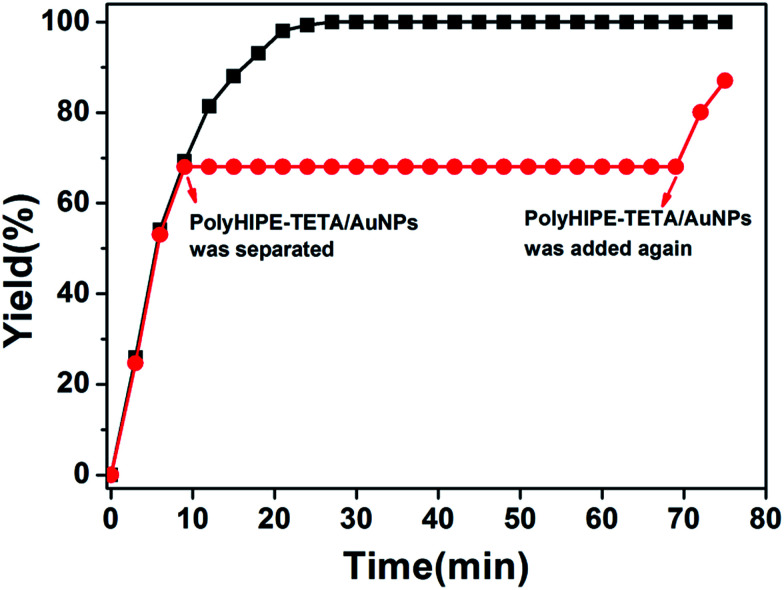
Catalytic activity of Au NPs in PHIPE–PGMA–TETA.

By observing the plot of yield against reaction time, we found that maximum yield was achieved in 24 min. In order to verify that almost no gold nanoparticles have fallen off the surface of PHIPE, we have conducted further experiments. As shown in Fig. 7, in ninth minutes, PHIPE–PGMA–TETA/Au NPs was separated, and the process of catalytic reaction stopped immediately. After 1 h, PHIPE–PGMA–TETA/Au NPs was added again, the catalytic reaction reoccurred. It was demonstrated that the reaction could be stalled and restarted by removing and restoring the catalyst, confirming the critical role of the Au NPs catalyst.

The reusable property of the monolith is important for the real application of catalysis. To investigate the reusable property of PHIPE–PGMA–TETA/Au NPs, the catalysis experiments were carried out repeatedly. In each cycle, the catalytic reduction was performed for 15 min; then, the catalyst was separated for the next cycle of catalysis. The separation of the catalyst was simple and convenient with tweezers, not as complex as other materials,^58–62^ as shown in Table S2 in ESI.† In the present work, the catalytic reaction was performed in a given time to investigate whether the conversion of the reaction was decreased to reveal the recovery and reusability of the catalyst.

The regeneration efficiency (RE) was defined as:
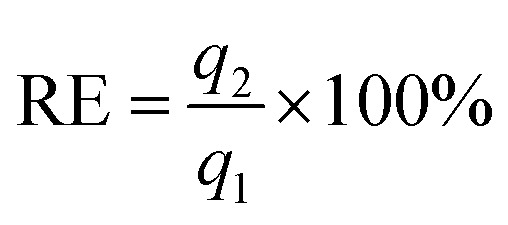
where *q*_1_ is the conversion of the reaction for the first use and *q*_2_ is that after a catalysis cycle operation.

**Fig. 8:**
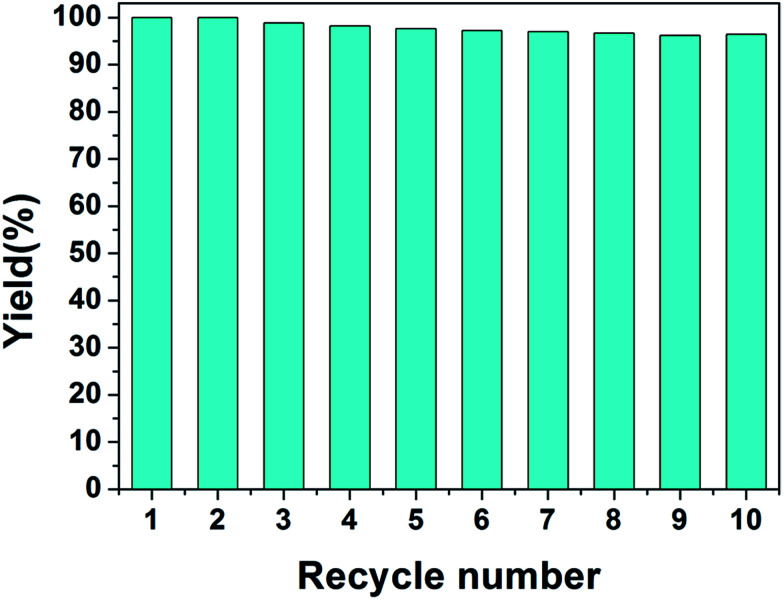
Repeated use of PHIPE–PGMA–TETA/Au NPs for the reduction of 4-NP.

Even after recycling for the tenth time, the conversion of 4-NP was only slightly decreased from 100% for the first cycle to 96.2%, as shown in Fig. 8. No weight loss of the monolith could be observed after the catalysis operation. The catalytic activity was well retained even after at least ten cycles of PHIPE–PGMA–TETA/Au NPs, suggesting the excellent immobilization ability of the grafted PGMA–TETA brush to gold nanoparticles as the catalyst.

## Conclusions

A simple way for the immobilization of gold nanoparticles on the surface of PHIPE for catalytic reaction was investigated. High internal phase emulsion polymerization was adopted for preparing macroporous polymeric monoliths PHIPE–Br. Through ATRP of glycidyl methacrylate, a dense and homogeneous coating of polymer was successfully grafted onto the surface of polyHIPE full of epoxy groups. Epoxy groups on the surface of PHIPE–PGMA could trigger ring-opening addition reaction with TETA. Gold nanoparticles could be easily *in situ* prepared and immobilized on the surface of amino-polymer modified functional macroporous PHIPE–PGMA–TETA. The catalytic materials PHIPE–PGMA–TETA/Au NPs have open-cell and porous structure, and can effectively catalyze the reduction of 4-nitrophenol. And they were recyclable without any decrease in activity, at least within 10 cycles. With post-polymerization methods, the polyHIPE surface could be easily decorated with all kinds of functional polymers, and immobilized with metal nanoparticles, thus, opening new possibilities of highly porous PHIPE for catalysis applications.

## Conflicts of interest

There are no conflicts to declare.

## Supplementary Material

RA-008-C8RA00089A-s001
