# Supplementary material for: Preparation and recyclable catalysis performance of functional macroporous polyHIPE immobilized with gold nanoparticles on its surface
Source: RSC Adv. 2018 Feb 6;8(11):5912–9. doi: 10.1039/c8ra00089a (PMC9078261; doi:10.1039/c8ra00089a)
Supplement: RA-008-C8RA00089A-s001 [file RA-008-C8RA00089A-s001.pdf]

Electronic Supplementary Material (ESI) for RSC Advances.
This journal is © The Royal Society of Chemistry 2018

Electronic Supplementary Information for:

Preparation and recyclable catalysis performance of functional macroporous polyHIPE immobilized with gold nanoparticles on its surface

Weizhong Yuana*, Xiangnan Chen[a], Yifan Xu[a], Chuan Yan[a], Yang Liu [a]*, Weishuai Lian[a]*, Yun Zhou[a]* and Zhihong Li[b]*

[a] *Tongji Hospital, School of Medicine, Department of Interventional and Vascular Surgeery of Shanghai Tenth People's Hospital, School of Materials Science and Engineering, Tongji University, Shanghai 201804, P. R. China. E-mail: yuanwz@tongji.edu.cn (W. Yuan), 25936615@qq.com (Y. Liu), lianweishuai@126.com (W. Lian), Zhouyunsy@126.com (Y. Zhou)*

[b] *Division of General Surgery, Shanghai Pudong New District Zhoupu Hospital, Shanghai 201200, P. R. China. E-mail: lance007@126.com (Z. Li)*

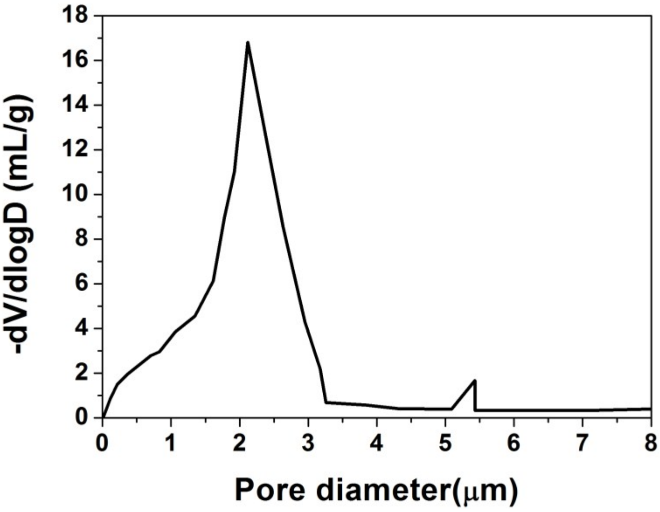

Fig. S1 Pore size distribution of PHIPE-PGMA-TETA/Au by MIP.

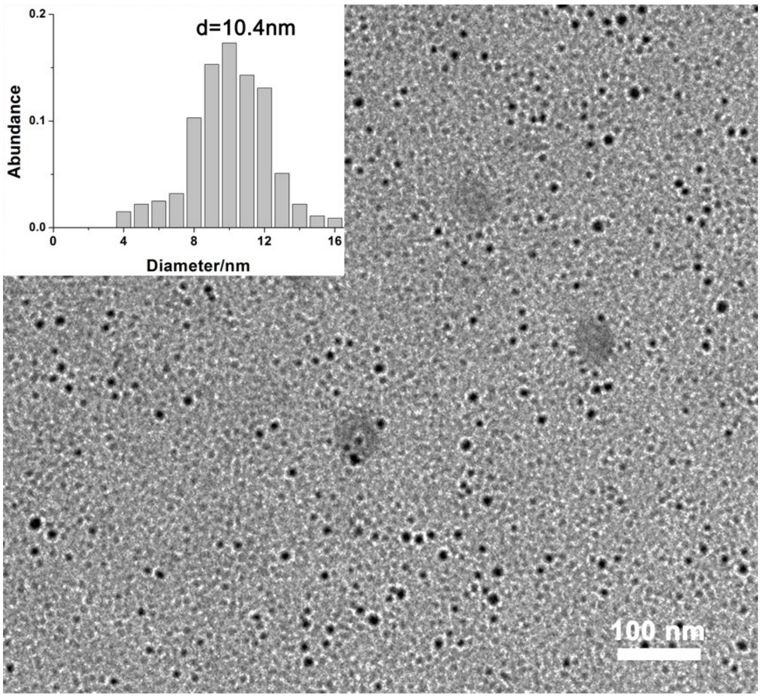

Fig. S2 TEM micrograph of Au NPs.

Table S1. Densities and porosities of PolyHIPEs

	PHIPE-PGMA-TETA	PHIPE-PGMA-TETA/Au
ρ^a, g cm^{-3}	0.2401	0.2882
δ^b, m^2 g^{-1}	43.2431	41.4738
V_g^c, ml g^{-1}	0.2496	0.2288
D^d, nm	25.63	26.24
$d_v \pm \sigma^e$, μm	2.3 ± 1.1	2.3 ± 1.0
P^f, %	86.7	86.4

[a] Dry polyHIPE density.

[b] Specific surface area from BET treatment of N_2 adsorption data.

[c] Pore volume from BET treatment of N_2 adsorption data.

[d] Average pore size within the walls of the monoliths and obtained from BJH treatment of N_2 adsorption data.

[e] Void diameter determined from SEM pictures of broken samples.

[f] Porosity of polyHIPE void structure from BET treatment of N_2 adsorption data.

Table S2. Comparison of the properties of nano-Au catalytic polymers based on the reduction of 4-Nitrophenol

Support	TOF(h^{-1})	Recyclable performance	Separation of materials	Reference
P4VP-g-CNC	3124.8	Not mentioned	Centrifugation	Carbohydrate Polymers 182 (2018) 61–68
Ionic cellulose	900	97%（5 times）	filtration with the addition of acetone	RSC Adv., 2018, 8, 1758–1763
HPEI-IBAm	120	95% (6 times)	Centrifugation	J. Mater. Chem., 2010, 20, 360–368
PEI/PVA nanofibers	<1	97% (3 times)	filtration	J. Mater. Chem., 2011, 21, 4493
magnetic porous carbon	35.4	>97%（6 times）	magnetic separation	J. Mater. Chem. A, 2014, 2, 18775–18785
PHIPE-PGMA-TETA.	6.3	>96%(10 times)	Tweezers out	This work
